# Langya henipavirus: Is it a potential cause for public health concern?

**DOI:** 10.1080/21505594.2022.2154188

**Published:** 2023-01-04

**Authors:** Shania Sanchez, Hinh Ly

**Affiliations:** Comparative and Molecular Biosciences Graduate Program, Department of Veterinary & Biomedical Sciences, College of Veterinary Medicine, University of Minnesota, St Paul, MN, USA

**Keywords:** Henipavirus, Langya virus, Nipah virus, Hendra virus, viral disease outbreaks

## Abstract

A new virus, named Langya henipavirus (LayV), has recently been identified in Shandong and Henan provinces in China and has so far infected 35 individuals between April 2018 and August 2021. It is closely related to other known henipaviruses (Nipah and Hendra viruses) that can cause up to 70% human case fatality. Even though LayV has not been shown to be fatal in humans and does not appear to be transmitted from human-to-human, it is an RNA virus with the capacity to evolve genetically in the infected hosts (e.g. shrews) and can infect humans (e.g. farmers who have been in close contacts with shrews). It is therefore important to be vigilant about this new viral outbreak.

A newly identified virus, named Langya henipavirus (LayV), has so far infected 35 individuals as of August 2021 in Shandong and Henan provinces in China (Figure 1), according to data provided by the Taiwan’s Centers for Disease Control, although it is unclear whether LayV is restricted to China. It can cause respiratory symptoms, such as fever, cough, and fatigue, but so far has not been associated with any human fatalities [1,2]. LayV has been classified, based on its genetic information, as a henipavirus that is closely related to only two known henipaviruses, i.e., Nipah virus and Hendra virus [3]. Unlike LayV, Nipah and Hendra viruses are known to cause fatal human diseases with case fatality ranging from 40% to 70% and therefore have been classified as risk group level 4 biological agents, because there are thus far no effective, FDA-approved vaccines [4] or treatments (besides supportive care to manage symptoms) for these deadly human viral pathogens. These henipaviruses are single-stranded negative-sense RNA viruses of the genus Henipavirus in the family *Paramyxoviridae* [4].

**Figure 1.**
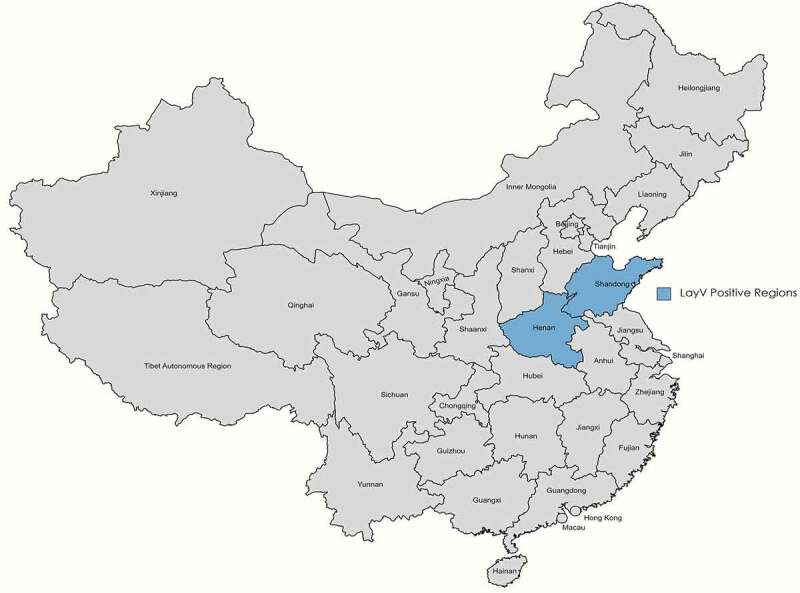
Shandong and Henan provinces in China (blue) show evidence of LayV infection in humans.

While bats, rodents, and shrews are thought to act as reservoirs for Nipah and Hendra viruses, a recent survey of more than two dozen wild species of animals suggests that shrews are the natural hosts of LayV [2], yet it isn’t clear whether they are primary or intermediate hosts of the virus [1,2], before it can be transmitted to people via direct contact with the infected animals. In fact, patients who have been tested positive for LayV are farmers who reported to have been in contact with shrews within a month of their symptom onsets. Symptoms of the disease include fever, fatigue, cough, loss of appetite, muscle pain, nausea, headache and vomiting [2]. Some patients have been diagnosed with more severe disease, such as liver and/or renal failure syndromes and/or decreased white blood cell count and low platelets [2]. Even though it has been reported that patients infected with LayV can shed viruses via blood, urine, faeces and saliva [5], so far, there is no evidence to suggest that LayV can be transmitted from human to human (but rather through zoonotic spillover events) (Figure 2).

**Figure 2.**
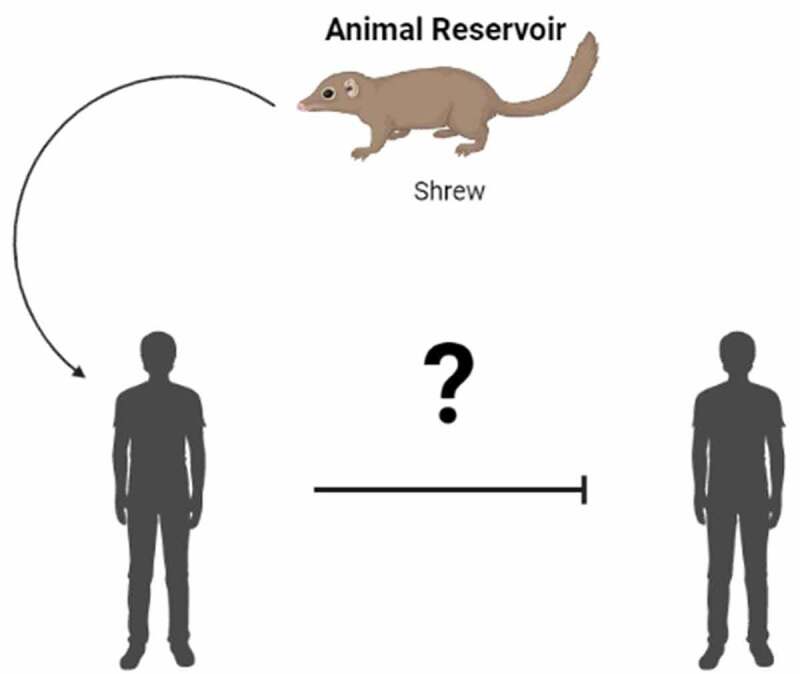
Transmission of LayV from shrew to human is known, but it is unclear whether human-to-human transmission of LayV is also possible.

Spillover of novel viruses, such as LayV and SARS-CoV-2, which is the causative viral agent of the current COVD-19 pandemic, has been strongly associated with close human interactions with animals (for a review, see [6]). Therefore, it is essential to understand how these events occurs to prevent future viral disease outbreaks and to minimize their societal impact [7].

While LayV is so far not an immediate cause for public health concern, it is an RNA virus with a relatively high degree of mutability (i.e., relatively high error rate of genomic RNA replication mediated by the error-prone RNA-dependent RNA polymerase [8]) and has the ability and potential to evolve genetically and to undergo rapid antigenic variations in the infected hosts, and therefore could become highly problematic if it is left unchecked. Therefore, it is essential that surveillance methods, such as those used during the 2018 outbreak of Nipah virus [9] that include serosurveys and longitudinal spatial and temporal studies to detect virus shedding and to isolate the virus from the likely animal reservoirs can help mitigate the potential threat that LayV may pose to humans.

Future investigations of the biology of LayV and of the host’s responses to this virus infection are needed to better understand this new virus-associated human disease. For example, based on published studies on Nipah and Hendra viruses, it might be possible to ascertain disease severity by monitoring the levels of pro-inflammatory cytokines [e.g. interleukins (IL-1, IL6, IL-8) and monocyte chemoattractant protein 1 (MCP-1) and colony stimulating factors (CSF)] released by virus-infected and/or uninfected cells [3,4], if it turns out that LayV infections may also cause a differential level of cytokine expressions. Additionally, it is important to understand how this new virus is transmitted and what host factors might restrict virus replication [3–5]. These efforts will aid in the development of effective prophylactic and/or therapeutic strategies against this new form of henipavirus infection.

## Data Availability

No primary data (figures and tables) are included in this article.
